# Mycorrhizal colonization and *Streptomyces viridosporus* HH1 synergistically up-regulate the polyphenol biosynthesis genes in wheat against stripe rust

**DOI:** 10.1186/s12870-023-04395-5

**Published:** 2023-08-10

**Authors:** Younes M. Rashad, Hany H. El-Sharkawy, Sara A. Abdalla, Omar M. Ibrahim, Nahla T. Elazab

**Affiliations:** 1 Plant Protection and Biomolecular Diagnosis Department, Arid Lands Cultivation Research Institute (ALCRI), City of Scientific Research and Technological Applications (SRTA-City), New Borg El-Arab City, Egypt; 2 Department of Mycology Research and Plant Diseases Survey, Plant Pathology Research Institute, Agricultural Research Center, Giza, Egypt; 3 Department of Plant Production, Arid Lands Cultivation Research Institute, City of Scientific Research and Technological Applications, Alexandria, Egypt; 4 Botany Department, Faculty of Science, Mansoura University, Mansoura, Egypt

**Keywords:** Chlorogenic acid, Flavonoids, Lignin, Mycorrhizal fungi, *Puccinia striiformis*, Resistance

## Abstract

**Background:**

Stripe rust is considered one of the most devastating diseases of wheat all over the world, resulting in a high loss in its production. In this study, time-course changes in expression of the polyphenol biosynthesis pathways genes in wheat against stripe rust were investigated. The defense mechanisms triggered by mycorrhizal colonization and/or spraying with *Streptomyces viridosporus* HH1 against this disease were also investigated.

**Results:**

Results obtained revealed that *C3H*, which is considered the key gene in lignin biosynthesis, was the most expressed gene. Furthermore, most of the chlorogenic acid and flavonoid biosynthesis genes were also overexpressed. Volcano plots of the studied genes reveal that the dual treatment led to a high significant overexpression of 10 out of the 13 studied genes. Heatmap of these genes showed that the most frequent expressed gene in response to all applied treatments along the study period was *DFR*, the key gene in the biosynthesis of anthocyanidins. Gene co-expression network of the studied genes showed that *HQT* was the most central gene with respect to the other genes, followed by *AN2* and *DFR*, respectively. Accumulation of different flavonoids and phenolic acids were detected in response to the dual treatment, in particular, cinnamic acid, coumarin, and esculetin, which recorded the highest elevation level recording 1000, 488.23, and 329.5% respectively. Furthermore, results from the greenhouse experiment showed that application of the dual treatment led to an 82.8% reduction in the disease severity, compared with the control treatment.

**Conclusions:**

We can conclude that the biosynthesis of lignin, chlorogenic acid, and flavonoids contributed to the synergistic triggering effect of the dual treatment on wheat resistance to stripe rust.

## Background

The cosmopolitan pathogenic fungus *Puccinia striiformis* f. sp. *tritici*, the causal agent of stripe rust, represents one of the highest devastating pathogens that threatens wheat crop (*Triticum aestivum* L.) worldwide. The annual global losses in wheat production due to stripe rust, commonly known as yellow rust, was estimated at around 6 million tons of wheat [1]. The disease appears as yellow to orange streaks of elongated pustules arranged in prominent stripes on the plant leaves and surrounded by chlorosis or necrosis area [2]. Once the uredospore is deposited on a wheat leaf, under favorable weather conditions, it needs few hours to germinate toward the leaf stomata. The grown mycelium penetrates the mesophyll cells and forms a balloon-shaped feeding structures, called haustoria, inside the cells. As the penetrating mycelium grows, a uredinium pustule is developed producing thousands of uredospores that can spread by wind repeating the infection many times through the field [3].

Different fungal and bacterial bio-agents have been studied for controlling various fungal plant diseases [4]. Among the studied bacterial biocontrol agents, members of the genus *Streptomyces* have the largest share in this regard. They can produce varied antagonistic bioactive metabolites such as antibiotics, siderophores, and lytic enzymes [5, 6]. In this concern, Rashad et al., [7] found that the antifungal activity of *S. griseorubens* E44G against the tomato wilt pathogen (*Fusarium oxysporum*) was attributed to its chitinolytic potential.

Arbuscular mycorrhizal fungi (AMF), phylum: Mucoromycota, subphylum: Glomeromycotina, are biotrophic symbionts that live in a mutualistic relationship with roots of the majority of the land plants [8]. One of the most crucial benefits achieved due to mycorrhizal colonization is induction of the host-defense responses against different attacking pathogens [9–11]. In this regard, multiple utilized mechanisms have been reported to be triggered due to the host mycorrhization including the cell wall thickening, cytoplasmic granulation, activation of pathogenesis-related (PR) proteins, induction of the transcriptomic expression of many defense-responsive genes, and accumulation of various fungitoxic polyphenolic compounds [4, 12].

The biosynthesis pathway of polyphenols is composed of three main metabolic routes: phenylpropanoid, flavonoid, and chlorogenic acid pathways. L-phenylalanine produced from the shikimate pathway in plastids represents the first step in the phenylpropanoid pathway, where it converts to *trans*-cinnamate by the action of the phenylalanine ammonia-lyase encoding gene (*PAL*). Using cinnamate 4-hydroxylase (*C4H*) and 4-coumaroyl CoA-ligase (*4CL*), *trans*-cinnamate can be converted to *p*-coumarate then *p*-coumaroyl-CoA, the central intermediate in the polyphenol biosynthesis pathway. *p*-coumaroyl-CoA can be converted to chlorogenic acid via the coumaroyl quinate route by hydroxycinnamoyl Co A: quinate hydroxycinnamoyl transferase (*HQT*), and *p*-coumarate-3-hydroxylase (*C3H*). Chlorogenic acid can also be produced via the coumaroyl shikimate route by hydroxycinnamoyl Co A: shikimate hydroxycinnamoyl transferase (*HCT*) [13]. For flavonoid biosynthesis, chalcone can be produced from *p*-coumaroyl-CoA by chalcone synthase (*CHS*). Chalcone is the structural precursor for many flavonoids and isoflavonoids [14]. By the action of chalcone isomerase (*CHI*), chalcone can be converted to flavanone, from which flavanones, flavones, and flavanols can be formed by the help of the flavanone 3-hydroxylase (*F3H*) and flavonoid 3’-hydroxylase (*F3’H*) encoding genes. In addition, flavonol synthase (*FLS*) and dihydroflavonol-4-reductase (*DFR*) encoding genes contribute in the flavonol and anthocyanin biosynthesis [15].

Based on a previous study that was conducted by the authors [16], it was found that mycorrhizal colonization of wheat plants and their spraying with *S. viridosporus* HH1 had an effective biocontrol effect against stripe rust disease on different defensive, physiological, growth, and ultrastructural levels. However, the triggered defensive mechanisms due to this treatment are not clear, particularly on the polyphenol biosynthesis pathways genes. Therefore, the current study aimed to investigate (1) the time-course transcriptomic patterns of the polyphenol biosynthesis pathways genes, (2) the polyphenol profiles, (3) the probable defensive modes of action, and (4) the disease severity in wheat plants infected with stripe rust in response to mycorrhizal colonization and/or treating with *S. viridosporus* HH1.

## Results

### Time-course transcriptomic patterns of the polyphenol biosynthesis pathways genes

**Fig. 1:**
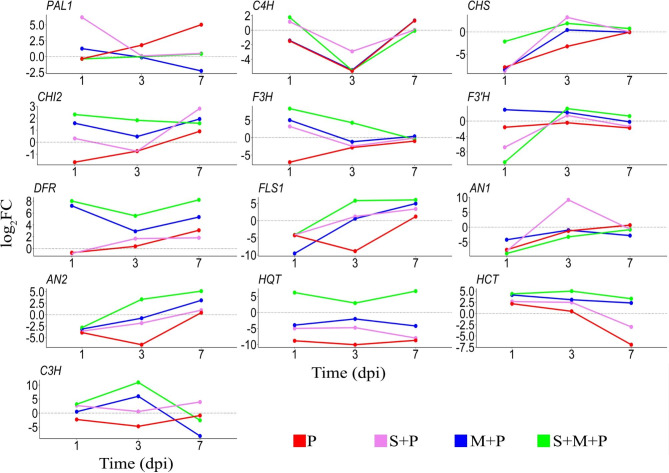
Line curves show the transcriptomic patterns of thirteen genes that regulate the polyphenol biosynthesis pathways in wheat leaves infected with stripe rust at 1, 3, and 7 dpi in response to mycorrhizal colonization and/or treating with *S. viridosporus* HH1. Where, P: infected and non-treated, M + P: infected and colonized with AMF, S + P: infected and sprayed with *S. viridosporus* HH1, and S + M + P: infected, colonized with AMF, and sprayed with *S. viridosporus* HH1. Each value is the mean of 3 biological replicates

**Fig. 2:**
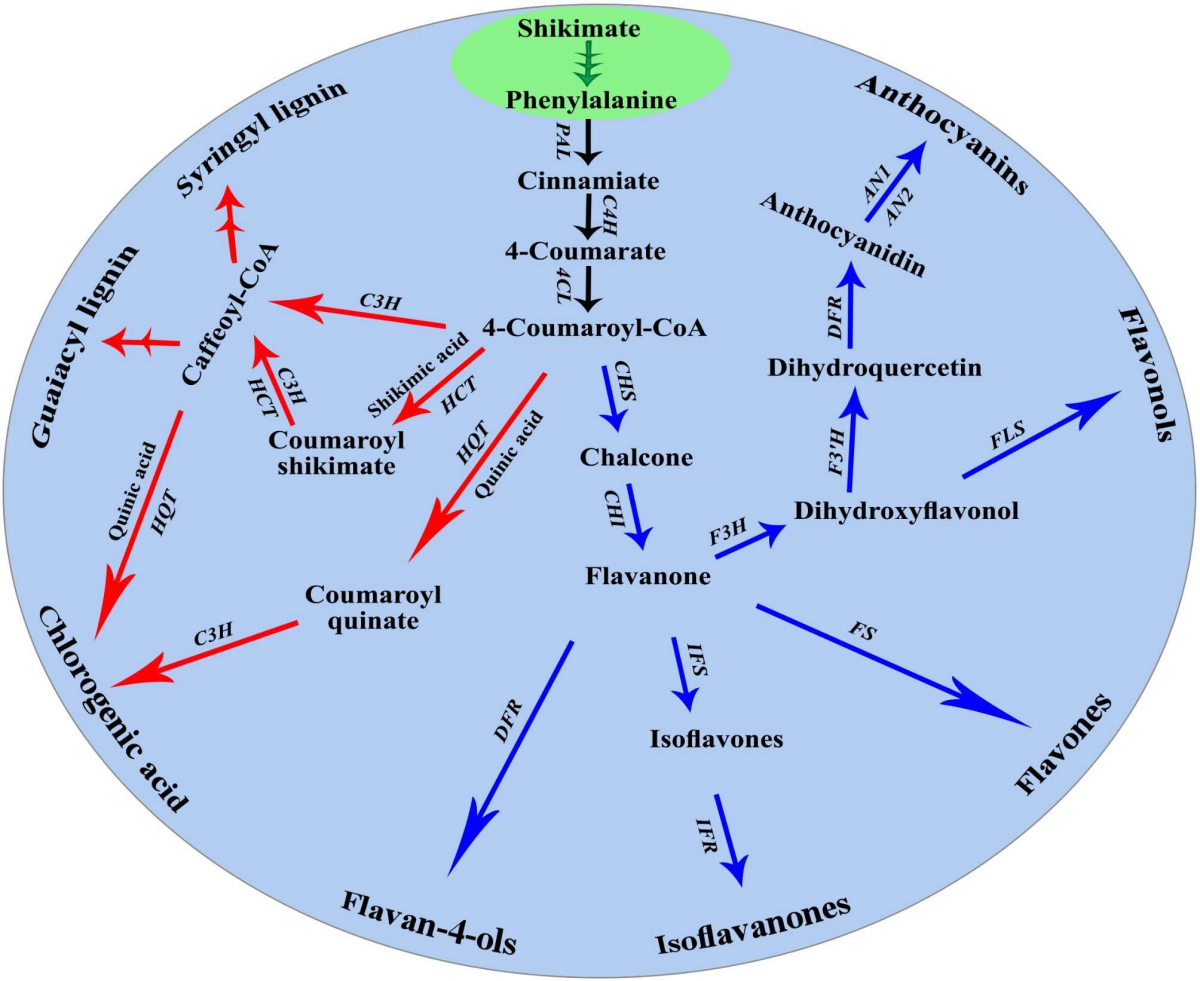
Pathway diagram of the polyphenol biosynthesis in the plant cell. Where, red arrows represent the chlorogenic acid pathway, blue arrows represent the flavonoid pathway, while arrows in black represent the phenylpropanoid pathway. *PAL*: phenylalanine ammonia lyase, *C4H*: cinnamic acid 4-hydroxylase, *4CL*: 4-coumarate-CoA-ligase, *CHS*: chalcone synthase, *CHI*: chalcone isomerase, *IFS*: isoflavone synthase, *IFR*: isoflavone reductase, *FS*: flavone synthase, *F3H*: flavanone 3-hydroxylase, *F3’H*: flavonoid 3’ hydroxylase, *DFR*: dihydroflavonol-4-reductase, *FLS*: flavonol synthase, *AN1*: anthocyanin 1, *AN2*: anthocyanin 2, HQT: hydroxycinnamoyl-CoA quinate hydroxycinnamoyl transferase, *C3H*: 4-coumarate 3-hydroxylase, and *HCT*: hydroxycinnamoyl-CoA shikimate hydroxycinnamoyl transferase (based on André et al. [38] and Falcone Ferreyra et al. [39])

Transcriptomic patterns of the polyphenol biosynthesis pathways genes in wheat leaves infected with stripe rust at 1, 3, and 7 days post infection (dpi) in response to mycorrhizal colonization and/or treating with *S. viridosporus* HH1 are illustrated in Fig. 1. In this study, expression of thirteen genes that regulate the three main parts of the polyphenol biosynthesis pathways: phenylpropanoid, flavonoid, and chlorogenic acid (Fig. 2) was investigated.

#### Time-course transcriptomic patterns in the phenylpropanoid pathway

Results obtained from the qPCR analysis indicated that infection of wheat plants with *P. striiformis* induced the expression level of *PAL1* in their leaves at 3 dpi. The expression level increased at 7 dpi (32-fold), while no effect was recorded at 1 dpi. Treatment (S + P) considerably upregulated *PAL1* at 1 dpi (73-fold), while no effect was observed at 3 and 7 dpi. While treatment (M + P) induced *PAL1* expression at 1 dpi but not at 3 dpi. A downregulation in the gene expression level was observed at 7 dpi. Application of the dual treatment (S + M + P) recorded no effect on *PAL1* expression at the three studied times. Regarding *C4H*, a downregulating effect was observed at 1 dpi due to infection with *P. striiformis*, this effect increased at 3 dpi, while at 7 dpi, an upregulation was recorded for this treatment. The same scenario was recorded for treatment (M + P). Treatments (S + P) and (M + P) triggered the expression of *C4H* at 1 dpi, while suppressed the gene expression at 3 dpi. No effect for both treatments was recorded at 7 dpi.

#### Time-course transcriptomic patterns in the flavonoid pathway

Data from Fig. 1 showed that all the applied treatments downregulated the expression level of *CHS* at 1 dpi. At 3 dpi, infection with stripe rust down regulated the gene expression, while treatments (S + P) or (S + M + P) induced the gene expression. No change in the *CHS* expression was observed for the treatment (M + P) at 3 dpi. No effect was recorded for all applied treatments on the gene expression at 7 dpi. For *CHI2*, no significant change was observed in the gene expression in wheat leaves due to the infection at 1, 3, and 7 dpi. Treatment (S + P) had no effect on the *CHI2* expression at 1 and 3 dpi, but upregulated the gene expression at 7 dpi (6.9-fold). Treatment (M + P) induced the expression level of *CHI2* at 1 and 7 dpi (3- and 3.7-fold, respectively), but not at 3 dpi. Application of the dual treatment (S + M + P) upregulated *CHI2* expression at 1, 3, and 7 dpi, recording 4.9-, 3.5-, and 3-fold, respectively. Regarding *F3H*, the gene expression was downregulated in wheat leaves due to infection with *P. striiformis* at 1 dpi, while no change was observed at 3 and 7 dpi. Results revealed that treatment (S + P) induced *F3H* expression level at 1 dpi (9.3-fold), but not at 3 and 7 dpi. While treatment (M + P) induced also the gene expression at 1 dpi (32.9-fold), but not at 3 and 7 dpi. A high upregulation in *F3H* expression was observed for the infected wheat plants, which treated with the dual treatment (S + M + P) at 1 and 3 dpi, recording 326- and 19.7-fold, respectively. While, no change was observed in the gene expression at 7 dpi. For *F3’H*, no change in the gene expression was recorded for wheat plants untreated-infected with stripe rust at all studied times. Treatment (S + P) led to a considerable downregulation in the expression level of *F3’H* at 1 dpi, and an upregulation at 3 dpi (2.7-fold), while no change was observed at 7 dpi. Treatment (M + P) resulted in induction of the gene expression at 1 and 3 dpi (7.7- and 4.7-fold, respectively), while no change in the gene expression was recorded at 7 dpi. The dual treatment (S + M + P) highly downregulated the expression of *F3’H* at 1 dpi, while this treatment led to an upregulation in the gene expression level at 3 and 7 dpi (9.4- and 2.4-fold, respectively). For *DFR*, the results obtained indicated that infection of wheat plants with stripe rust had no effect on the gene expression in their leaves at 1 and 3 dpi, but upregulated the gene expression at 7 dpi (8.5-fold). No change in the expression of *DFR* was recorded for the treatment (S + P) at 1 dpi, while an upregulation in the gene expression was observed at 3 and 7 dpi (3.2- and 3.5-fold, respectively). The treatment (M + P) induced the *DFR* expression at 1, 3, and 7 dpi (148.7-, 7.5-, and 40.1-fold, respectively). The dual treatment (S + M + P) also considerably triggered the expression level of *DFR* at 1, 3, and 7 dpi, recording 260.5-, 46.1-, and 297.7-fold, respectively. For *FLS1*, infection of wheat plants decreased the gene expression at 1 and 3 dpi, but induced it at 7 dpi (2.3-fold). Treatment (S + P) downregulated the gene expression at 1 dpi, but upregulated it at 3 and 7 dpi (2.3-, and 10.7-fold, respectively). Application of treatment (M + P) led to suppression of the *FLS1* expression at 1 dpi, and induced the expression at 7 dpi (31.8-fold), while did not affect the gene expression at 3 dpi. Application of the dual treatment (S + M + P) downregulated the *FLS1* expression at 1 dpi, but induced it at 3 and 7 dpi, recording 57.2- and 67-fold, respectively. For *AN1*, all treatments suppressed the gene expression at 1 dpi. At 3 dpi, no effect was observed for all treatment, except (S + P) which highly triggered the gene expression (573-fold). At 7 dpi, no change in the gene expression was observed for all applied treatments. For *AN2*, all studied treatments inhibited the gene expression at 1 dpi. At 3 dpi, treatments (P) and (S + P) downregulated the gene expression, and the dual treatment (S + M + P) upregulated it (10.1-fold). No effect was recorded for the treatment (M + P). At 7 dpi, the treatments (P) and (S + P) had no effect on the gene expression, while the treatments (M + P) and (S + M + P) upregulated the gene expression (8.6- and 34.6-fold, respectively).

#### Time-course transcriptomic patterns in the chlorogenic acid pathway

Three genes (*HQT*, *HCT*, and *C3H*) were studied in this pathway. Regarding *HQT*, application of the three treatments (P), (S + P), and (M + P) led to a downregulation of the gene expression at all studied times. In contrast, application of the dual treatment (S + M + P) upregulated the expression of this gene, recording 73-, 7.5-, and 99.4-fold, respectively. For *HCT*, all applied treatments induced the gene expression at 1 and 3 dpi, except for the treatment (P) at 3 dpi which had no effect on the gene expression. At 7 dpi, both treatments (P) and (S + P) downregulated the gene expression, while treatments (M + P) and (S + M + P) triggered the gene expression (5.2- and 9.8-fold, respectively). For *C3H*, treatments (S + P) and (S + M + P) induced the gene expression (6.5-and 8.8-fold, respectively) at 1 dpi, while no change in the gene expression was observed for the treatments (M + P) and (P). At 3 dpi, the treatment (P) downregulated the gene expression, while the treatment (S + P) had no effect in this regard. In contrast, application of the treatment (M + P) and the dual treatment (S + M + P) led to a high upregulation in the *C3H* expression, recording 64- and 1977-fold, respectively. At 7 dpi, application of the treatment (S + P) induced the gene expression (15.5-fold), while the treatment (M + P) downregulated it. No change in the gene expression was observed for the treatments (P) and (S + M + P).

### Statistical significance of the differential gene expression

**Fig. 3:**
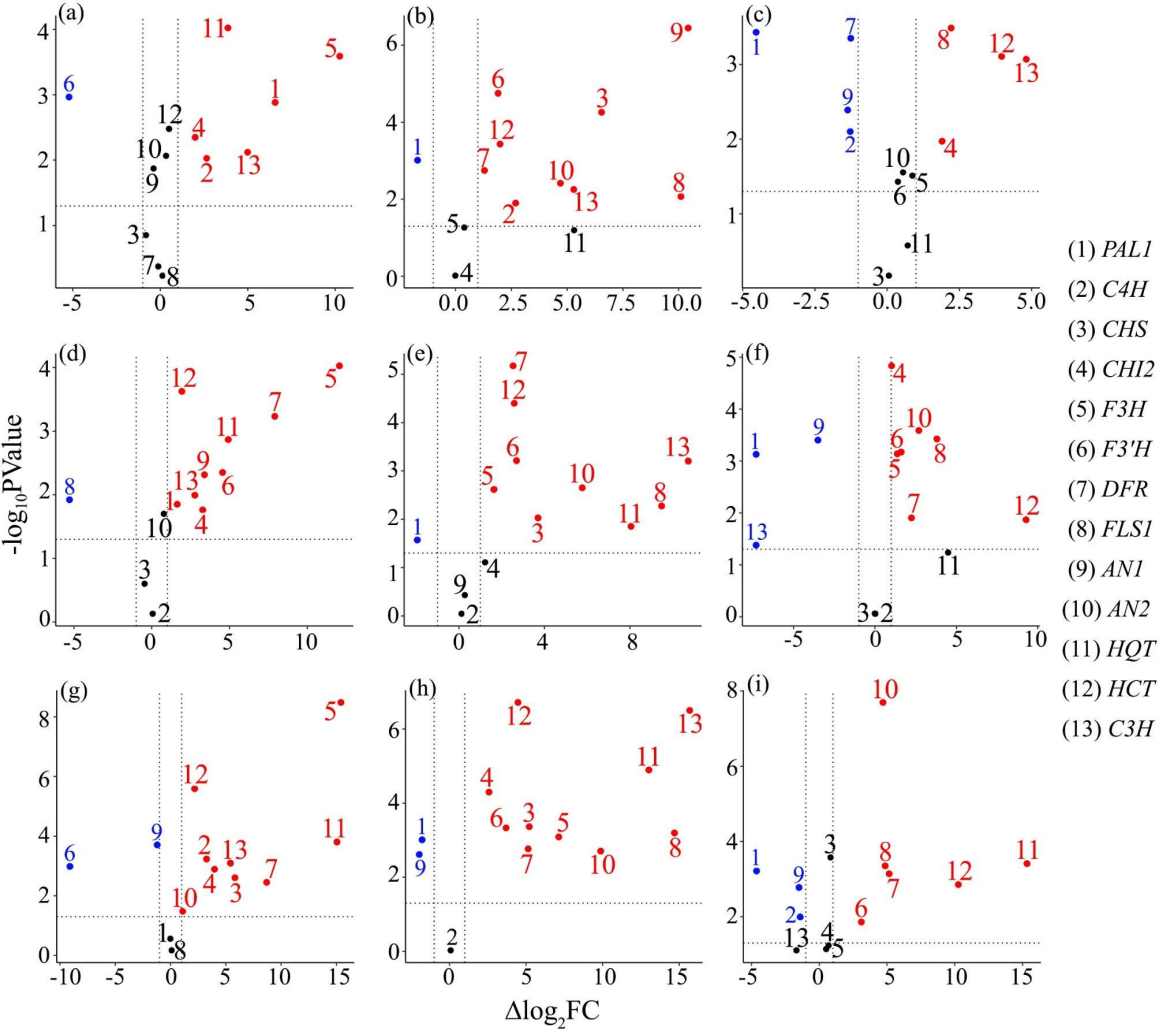
Volcano plots of the polyphenol biosynthesis pathways genes in wheat leaves infected with stripe rust showing the significance and magnitude of change in the genes expression in response to the applied treatments, compared to the nonmycorrhizal-untreated-infected treatment. Where (**a**) infected plants and sprayed with *S. viridosporus* HH1 (S + P) at 1 dpi, (**b**) treatment (S + P) at 3 dpi, (**c**) treatment (S + P) at 7 dpi, (**d**) infected plants and colonized with AMF (M + P) at 1 dpi, (**e**) treatment (M + P) at 3 dpi, (**f**) treatment (M + P) at 7 dpi, (**g**) mycorrhizal-infected plants and sprayed with *S. viridosporus* HH1 (S + M + P) at 1 dpi, (**h**) treatment (S + M + P) at 3 dpi, and (**i**) treatment (S + M + P) at 7 dpi. Red numbers represent the upregulated genes; blue numbers represent the downregulated genes, while numbers in black represent the unaffected genes

Volcano plots were generated for all studied genes to investigate the statistical significance of the fold change in the gene expression in response to different applied treatments along the study period, compared to the untreated-infected wheat plants (Fig. 3). For the treatment (S + P) at 1 dpi, the volcano plot revealed that six genes were significantly upregulated (*PAL1*, *C3H*, *C4H*, *CHI2*, *HQT* and *F3H*). Among them, *HQT* and *F3H* showed a very high significant upregulation (*p* ≤ 0.001). One gene (*F3’H*) was significantly downregulated, while expression of six genes unchanged, compared to the untreated-infected wheat plants (Fig. 3a). For the treatment (S + P) at 3 dpi, the volcano plot showed that nine genes were significantly upregulated (*C4H*, *CHS*, *F3’H, DFR, FLS1, AN1, AN2, HCT*, and *C3H*). Among them, *AN1*, *CHS*, *HCT*, and *F3’H* showed a very high significant upregulation (*p* ≤ 0.001). In contrast, one gene (*PAL1*) showed a very high significant downregulation (*p* ≤ 0.001), while three genes showed unchanged expression, compared to the untreated-infected wheat plants (Fig. 3b). For the treatment (S + P) at 7 dpi, expression of four genes (*CHI2*, *FLS1, HCT*, and *C3H*) exhibited a significant upregulation. Among them, *FLS1, HCT*, and *C3H* showed a very high significant elevation in their expression (*p* ≤ 0.001). Four genes showed a significant down regulation (*PAL1*, *C4H*, *DFR*, and *AN1*). Among them, two genes (*PAL1* and *DFR*) showed a very high significant downregulation (*p* ≤ 0.001). The remaining 5 genes did not show any significant change in their expression, compared to the control treatment (P) as illustrated in Fig. (3c).

Volcano plot (Fig. 3d) demonstrated that mycorrhizal colonization of the infected wheat plants (M + P) at 1 dpi led to a significant upregulation of nine genes (*PAL1, CHI2, F3H, F3’H, DFR, AN1, HQT, HCT*, and *C3H*). Three of these genes (*F3H, DFR*, and *HCT*) showed a very high significant upregulation (*p* ≤ 0.001). On the contrary, one gene (*FLS1*) was significantly downregulated, while three other genes showed no change in their expression, compared to the control treatment (P). For treatment (M + P) at 3 dpi, results from the volcano plot (Fig. 3e) indicated that nine of the studied genes (*CHS*, *F3H, F3’H, DFR, FLS1, AN2, HQT, HCT*, and *C3H*) were significantly upregulated due to mycorrhizal colonization of the infected wheat plants, compared to the nonmycorrhizal-untreated-infected plants (P). Four of these genes (*C3H*, *DFR, F3’H*, and *HCT*) exhibited a very high significant upregulation (*p* ≤ 0.001). One gene (*PAL1*) showed a significant downregulation, while three genes showed an unchanged expression, compared to the control treatment (P). Volcano plot of treatment (M + P) at 7 dpi (Fig. 3f) showed that seven of the studied genes (*CHI2*, *F3H, F3’H, DFR, FLS1, AN2*, and *HCT*) were significantly upregulated due to this treatment. All of them, except *DFR* and *HCT*, showed a very high significant upregulation (*p* ≤ 0.001). In addition, three of the studied genes (*PAL1, AN1*, and *C3H*) were significantly downregulated in response to this treatment. In this regard, *PAL1* and *AN1* exhibited a very high significant downregulation (*p* ≤ 0.001). Expression of three other genes was unchanged.

Volcano plot of the dual treatment (S + M + P) at 1 dpi (Fig. 3g) revealed that nine of the studied genes (*C4H*, *CHS*, *CHI2*, *F3H, DFR, AN2, HQT, HCT*, and *C3H*) were significantly upregulated due to spraying of the mycorrhizal-infected wheat plants with *S. viridosporus* HH1, compared to the treatment (P). Five of these nine genes (*C4H*, *F3H, HQT, HCT*, and *C3H*) showed a very high significant upregulation (*p* ≤ 0.001). In addition, two genes (*F3’H*, and *AN1*) were significantly downregulated, while two other genes showed unchanged expression, compared to the control treatment (P). For treatment (S + M + P) at 3 dpi, volcano plot (Fig. 3h) demonstrated that ten of the thirteen genes included in this study (*CHS*, *CHI2*, *F3H, F3’H, DFR, AN2, HQT, HCT, FLS1* and *C3H*) were significantly upregulated due to this treatment. All of the ten genes, except *DFR* and *AN2*, showed a very high significant upregulation (*p* ≤ 0.001). In addition, two genes (*PAL1* and *AN1*) were significantly downregulated, while one gene showed an unchanged expression, compared to the control treatment (P). Volcano plot (Fig. 3i) revealed that six genes (*F3’H, DFR, FLS1, AN2, HQT*, and *HCT*) were significantly upregulated due to the dual treatment (S + M + P) at 7 dpi. Four of these genes (*DFR, FLS1, AN2*, and *HQT*) exhibited a very high significant upregulation (*p* ≤ 0.001). Furthermore, three genes (*PAL1, C4H*, and *AN1*) were significantly downregulated due to this treatment, while four genes were unaffected, compared to the control treatment (P).

### Hierarchical clustering of the differentially expressed genes

**Fig. 4:**
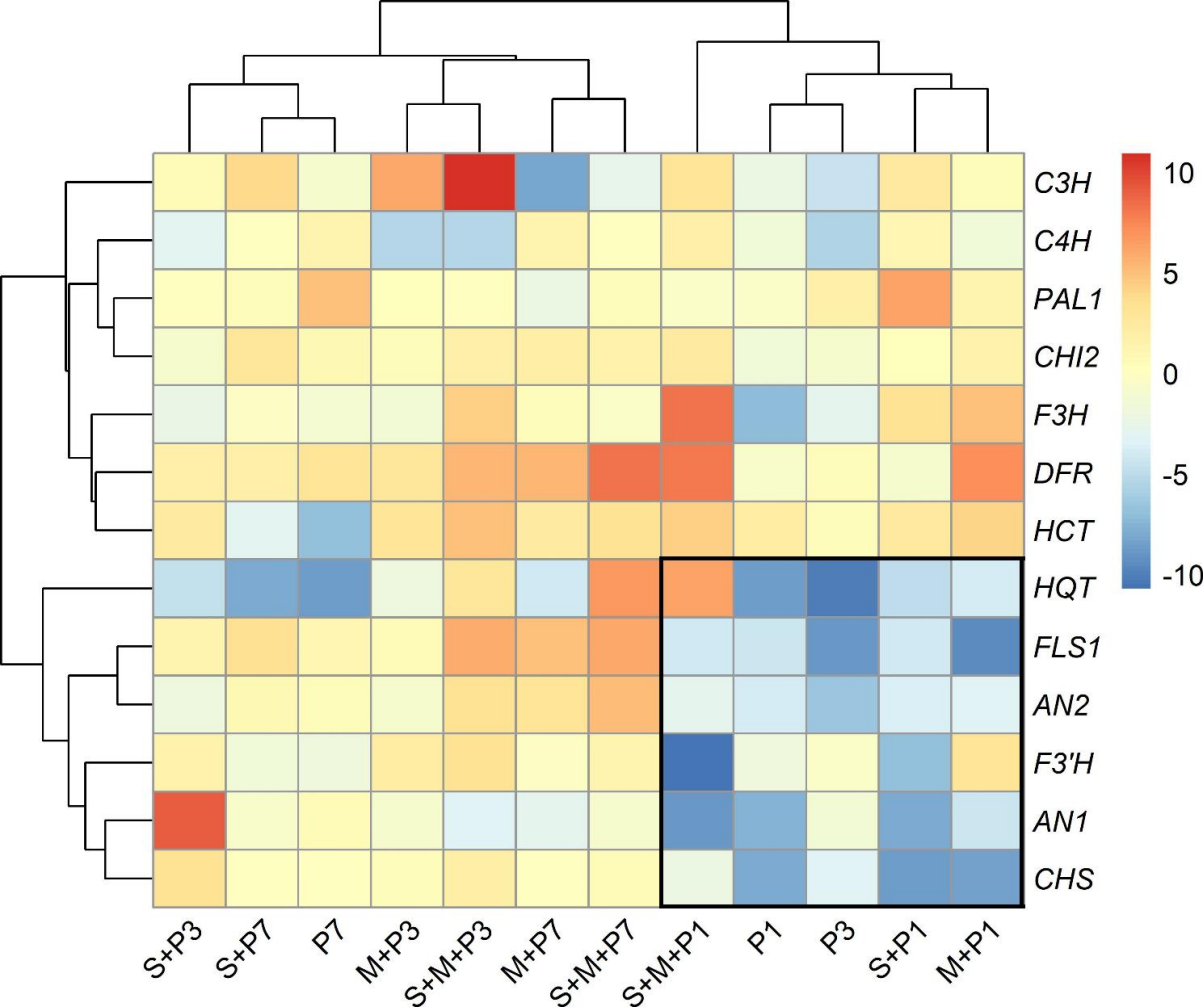
Hierarchical cluster heatmap shows degree of the transcriptomic expression of the polyphenol biosynthesis pathways genes in wheat leaves infected with stripe rust at 1, 3, and 7 dpi in response to mycorrhizal colonization and/or treating with *S. viridosporus* HH1. Where, P: infected and non-treated, M + P: infected and colonized with AMF, S + P: infected and sprayed with *S. viridosporus* HH1, and S + M + P: infected, colonized with AMF, and sprayed with *S. viridosporus* HH1

Heatmap of the studied genes in wheat leaves showing their differential expression in response to the applied treatments along the study period is illustrated in Fig. 4. The hierarchical cluster analysis showed that all the applied treatments at different times are clustered in two main clusters. The first one includes all treatments at 1 dpi, in addition to treatment (P) at 3 dpi (early stage), while the other main cluster includes the remaining treatments at 3 and 7 dpi (late stage). The first main cluster is divided into two sub-clusters; one of them includes a single treatment (S + M + P) at 1 dpi, while the other is divided into two sub-sub-clusters. Treatments (S + P) and (M + P) at 1 dpi are clustered in one sub-sub-cluster, while treatments (P) at 1 and 3 dpi are clustered in the other one. The second main cluster is divided into two sub-clusters; each of them includes four treatments in two sub-sub-clusters. In the first sub-sub-cluster, treatments (M + P) and (S + M + P) at 3 dpi are clustered, while the other sub-sub-cluster includes (M + P) and (S + M + P) at 7 dpi. The second sub-cluster includes three treatments in two sub-sub-clusters. One sub-sub-cluster includes (S + P) and (P) at 7 dpi, while the second sub-sub-cluster includes one single treatment (S + P) at 3 dpi.

Regarding the studied genes, the hierarchical cluster analysis revealed that all genes are clustered in two main clusters; seven genes in one and six genes in the other one. The first main cluster includes three sub-clusters; one of them contains (*C4H*, *PAL1*, and *CHI2*), the second sub-cluster contains (*F3H*, *DFR*, and *HCT*), while the third one contains one gene (*C3H*). The second main cluster includes two sub-clusters; one of them contains five genes (*FLS1*, *AN2*, *F3’H*, *AN1*, and *CHS*), while the other includes one gene (*HQT*). Data obtained showed that all treatments at 1 dpi, in addition to treatment (P) at 3 dpi, led to a downregulation, at varied degrees, of six genes in the polyphenol synthetic pathways (*HQT*, *FLS1*, *AN2*, *F3’H*, *AN1*, and *CHS*) (the black square). In contrast, the heatmap showed that the dual treatment (S + M + P) at 3 and 7 dpi led to upregulation of the majority of the studied genes. The most frequent expressed gene in response to all applied treatments along the study period was *DFR*, while the highest upregulated gene was *C3H* in response to the dual treatment (S + M + P) at 3 dpi.

### Correlations between the studied genes

Values of the pairwise correlation between the polyphenol biosynthesis pathways genes in wheat leaves infected with stripe rust in response to the applied treatments are presented in Table 1. Data obtained demonstrated many significant correlations between them, at varied degrees. Results in Table 1 showed that *AN2*-*FLS1*, *HQT-DFR*, and *HQT-HCT* had positive very-high-significant correlations (*p* ≤ 0.001). In addition, *AN2-HQT, AN2-DFR, F3H-CHI2, F3H-DFR, FLS1-CHS*, and *AN1-CHS* had a positive high-significant correlation (*p* ≤ 0.01), while *F3’H-C4H* showed a negative high-significant correlation (*p* ≤ 0.01). Furthermore, various positive significant pairwise-correlations (*p* ≤ 0.05) were also recorded such as *AN2-CHS, AN2-CHI2, F3’H-CHS, F3’H-HCT, F3H-HCT, F3H-HQT, FLS1-DFR*, and *DFR- CHI2.* In contrast, *F3H-AN1* and *HCT-AN1* exhibited a negative significant correlation (*p* ≤ 0.05). The other pairs showed no significant correlation with each other.

**Table 1:** Kendall’s Tau rank correlations (r) matrix between the polyphenol biosynthetic pathways genes in wheat leaves infected with stripe rust in response to the applied treatments

	*C4H*	*AN1*	*PAL1*	*C3H*	*CHS*	*CHI2*	*DFR*	*HCT*	*HQT*	*FLS1*	*F3H*	*F3’H*	*AN2*
*C4H*	1.000	-0.121	0.057	-0.161	-0.245	0.254	0.142	-0.125	-0.002	0.057	0.296	-0.530**	0.076
*AN1*		1.000	0.117	-0.017	0.442**	-0.117	-0.051	-0.345*	-0.131	0.171	-0.402*	0.129	0.121
*PAL1*			1.000	-0.002	-0.245	-0.220	-0.226	-0.197	-0.252	-0.220	-0.034	-0.246	-0.224
*C3H*				1.000	0.178	0.157	0.042	0.324	0.258	0.040	0.264	0.146	0.138
*CHS*					1.000	0.059	0.224	0.108	0.269	0.502**	-0.112	0.408*	0.400*
*CHI2*						1.000	0.427*	0.174	0.283	0.288	0.534**	0.004	0.413*
*DFR*							1.000	0.305	0.550***	0.389*	0.446**	0.305	0.499**
*HCT*								1.000	0.620***	0.091	0.413*	0.413*	0.201
*HQT*									1.000	0.339	0.400*	0.301	0.442**
*FLS1*										1.000	0.133	0.224	0.792***
*F3H*											1.000	0.000	0.273
*F3’H*												1.000	0.265
*AN2*													1.000

Values followed by *, ** or *** are significant at *p* ≤ 0.05, *p* ≤ 0.01 or *p* ≤ 0.001, respectively

**Fig. 5:**
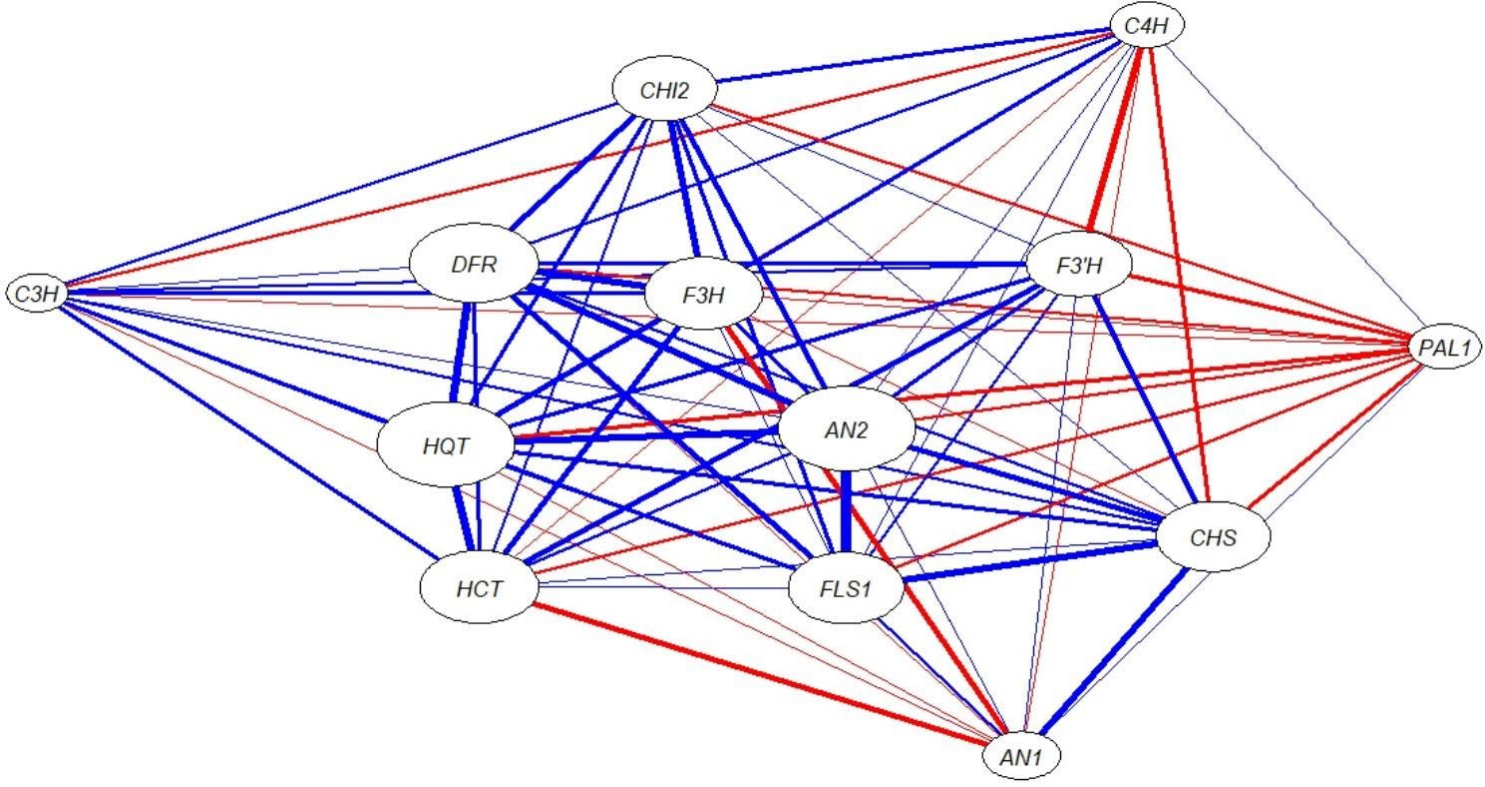
Gene co-expression network shows the correlations between the polyphenol biosynthesis pathways genes in wheat leaves infected with stripe rust in response to mycorrhizal colonization and/or treating with *S. viridosporus* HH1. Where the blue edges represent the positive correlations, while the red edges represent the negative correlations. The edge thickness represents the correlation degree between two genes, and length of the edge represents the degree of separation between the nodes pair, while the node size represents the degree of centrality of the gene

The gene co-expression network, based on Kendall’s Tau rank correlation matrix, is illustrated in Fig. 5. Results obtained from the gene co-expression network show that *HQT* was the most central gene with regard to the other genes, which means that it had the highest number and degree of significant correlations with the other genes, followed by *AN2*, and *DFR*, respectively. On the contrary, *C3H* and *PAL1* were peripheral in the co-expression network showing no significant correlation with the other genes. Furthermore, the gene co-expression network reveals that the genes *DFR, HQT, HCT, F3H*, *F3’H*, *AN2, CHS*, and *FLS1* form one community, which means that they have positive significant interrelationships with each other a way from *C3H, AN1, PAL1*, and *C4H*.

### Disease severity and infection type

**Fig. 6:**
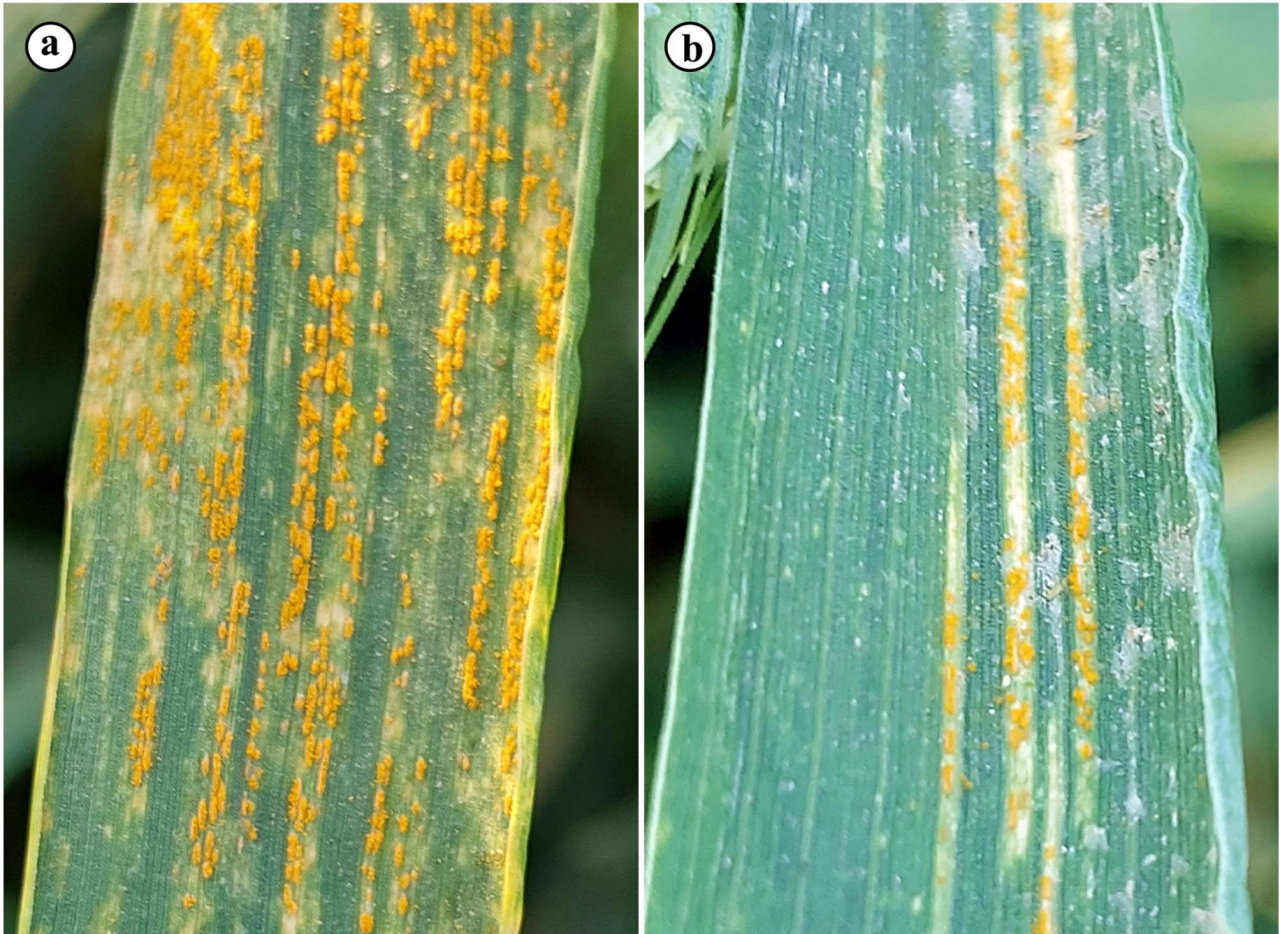
Photographs show severity of stripe rust in wheat leaves, where (a) untreated-infected leaf and (b) infected leaf and treated with mycorrhizal fungi and *S. viridosporus* HH1

Disease severity of stripe rust in wheat plants in response to colonization with AMF and/or treating with *S. viridosporus* HH1 two weeks after infection is presented in Table 2; Fig. 6. Results from the greenhouse experiment indicated that all applied treatments significantly reduced the disease severity, compared to the nonmycorrhizal-untreated-infected plants. No significant difference was observed between the single treatments (S + P) and (M + P), while the least severity was recorded for the dual treatment (S + M + P) recording 16.6% and infection type (MR), compared with the nonmycorrhizal-untreated-infected treatment (P), which recorded 96.7% and infection type (S).

**Table 2:** Disease severity of stripe rust in wheat plants in response to colonization with AMF and/or treating with *Streptomyces viridosporus* HH1 two weeks after infection

Treatment	Disease severity (%)*	Infection type
P	96.7 ± 2.9^a^	S
S + P	45.3 ± 3.3^b^	MS
M + P	32.5 ± 2.7^b^	MR
S + M + P	16.6 ± 2.1^c^	MR

* Values followed by the same letter are not significantly different according to Tukey’s HSD test (*P* ≤ 0.05), each value is the mean of five replicates ± SD. Where, P: infected and non-treated, M + P: infected and colonized with AMF, S + P: infected and sprayed with *S. viridosporus* HH1, and S + M + P: infected, colonized with AMF, and sprayed with *S. viridosporus* HH1, S: susceptible, MS: moderately susceptible, and MR: moderately resistant

### HPLC profiling of the flavonoids and phenolic acids

**Fig. 7:**
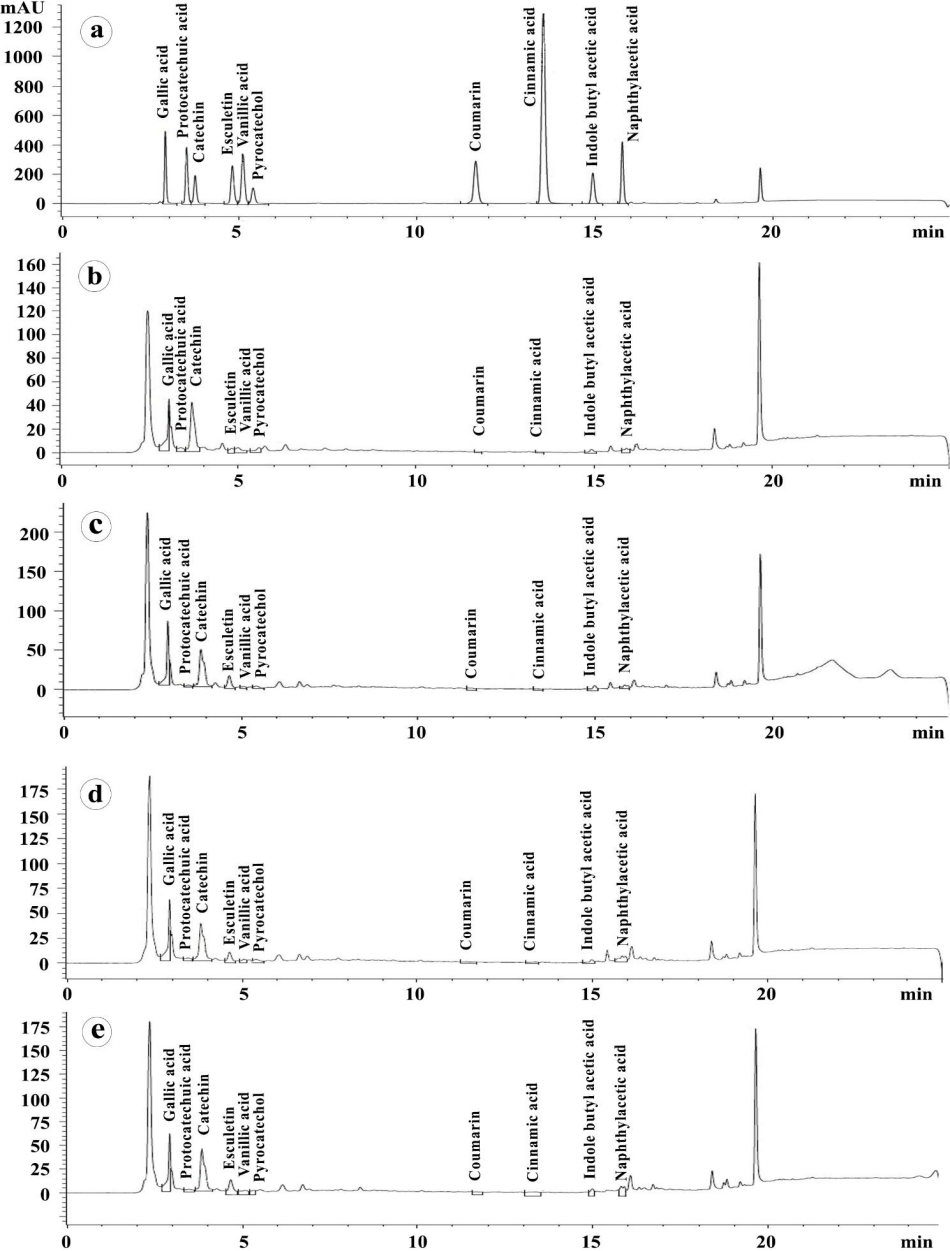
HPLC chromatograms show the flavonoid profiles in leaves of wheat plants infected with stripe rust in response to mycorrhizal colonization and/or treating with *S. viridosporus* HH1 at 7 dpi. Where, (**a**) mixture standard solution of different flavonoids and phenolic acids, (**b**) nonmycorrhizal-infected-untreated wheat plants (P), (**c**) nonmycorrhizal-infected wheat plants, which were sprayed with *S. viridosporus* HH1 (S + P), (**d**) mycorrhizal-infected-untreated wheat plants (M + P), and (**e**) mycorrhizal-infected wheat plants, which sprayed with *S. viridosporus* HH1 (S + M + P)

HPLC profiles of some flavonoids and phenolic acids in wheat leaves infected with stripe rust in response to colonization with AMF and/or treating with *S. viridosporus* HH1 at 7 days after infection are presented in Table 3 and illustrated in Fig. 7. Results from the HPLC analysis revealed that all the estimated flavonoids and phenolic acids, except pyrocatechol, significantly increased in leaves of the infected wheat plants colonized with AMF and sprayed with *S. viridosporus* HH1 (S + M + P), compared to the nonmycorrhizal-untreated-infected plants (P). Furthermore, the single treatments (S + P) and (M + P) led to a significant accumulation of most of the analyzed polyphenolic compounds, compared with the control treatment (P). However, the increment in their concentrations due to the dual treatment (S + M + P) mostly was higher than that due to the single treatments (S + P) and (M + P). The highest increment in accumulation of the analyzed compounds was recorded for cinnamic acid (1000%), followed by coumarin (488.23%) and esculetin (329.5%), while the least one was recorded for pyrocatechol (11.32%), compared with the control treatment (P). On the contrary, the analysis data showed that the single treatments (S + P) and (M + P) led to a decrease in, or not affected, vanillic acid and protocatechuic acid concentrations, compared with the treatment (P).

**Table 3:** HPLC profiling of some polyphenolic compounds (mg mL^− 1^) in wheat leaves infected with stripe rust in response to the applied treatments at 7 days after infection

Name	Retention Time	Treatments			
		P	S + P	M + P	S + M + P
Gallic acid	2.989	4.81 ± 0.70^c^	7.77 ± 0.28^a^	7.25 ± 0.08^b^	7.25 ± 0.12^b^
Protocatechuic acid	3.496	1.73 ± 0.12^b^	1.36 ± 0.09^c^	1.64 ± 0.07^b^	2.12 ± 0.06^a^
Catechin	3.737	39.36 ± 1.47^d^	50.26 ± 0.59^a^	43.04 ± 1.06^c^	46.88 ± 1.2^b^
Esculetin	4.783	2.00 ± 0.80^d^	7.05 ± 0.68^b^	5.02 ± 0.23^c^	8.59 ± 0.13^a^
Vanillic acid	5.080	2.26 ± 0.21^b^	0.94 ± 0.30^d^	1.29 ± 0.15^c^	2.72 ± 0.10^a^
Pyrocatechol	5.367	2.56 ± 0.07^b^	2.38 ± 0.05^c^	2.85 ± 0.04^a^	2.50 ± 0.03^b^
Coumarin	11.627	0.17 ± 0.03^c^	0.76 ± 0.08^b^	0.68 ± 0.01^b^	1.00 ± 0.04^a^
Cinnamic acid	13.528	0.09 ± 0.03^c^	0.29 ± 0.04^b^	0.27 ± 0.02^b^	0.99 ± 0.06^a^
4,3-indole butyl acetic acid	14.922	2.16 ± 0.29^d^	4.54 ± 0.04^a^	2.67 ± 0.13^c^	3.55 ± 0.03^b^
Naphthylacetic acid	15.750	1.68 ± 0.02^d^	2.25 ± 0.02^c^	3.91 ± 0.04^a^	3.37 ± 0.02^b^

* In each row, values followed by the same letter are not significantly different according to Tukey’s HSD test (*P* ≤ 0.05), each value is the mean of three replicates ± SD. Where, P: infected and non-treated, S + P: infected and sprayed with *S. viridosporus* HH1, M + P: infected and colonized with AMF, and S + M + P: infected, colonized with AMF, and sprayed with *S. viridosporus* HH1

### Colonization with AMF

**Fig. 8:**
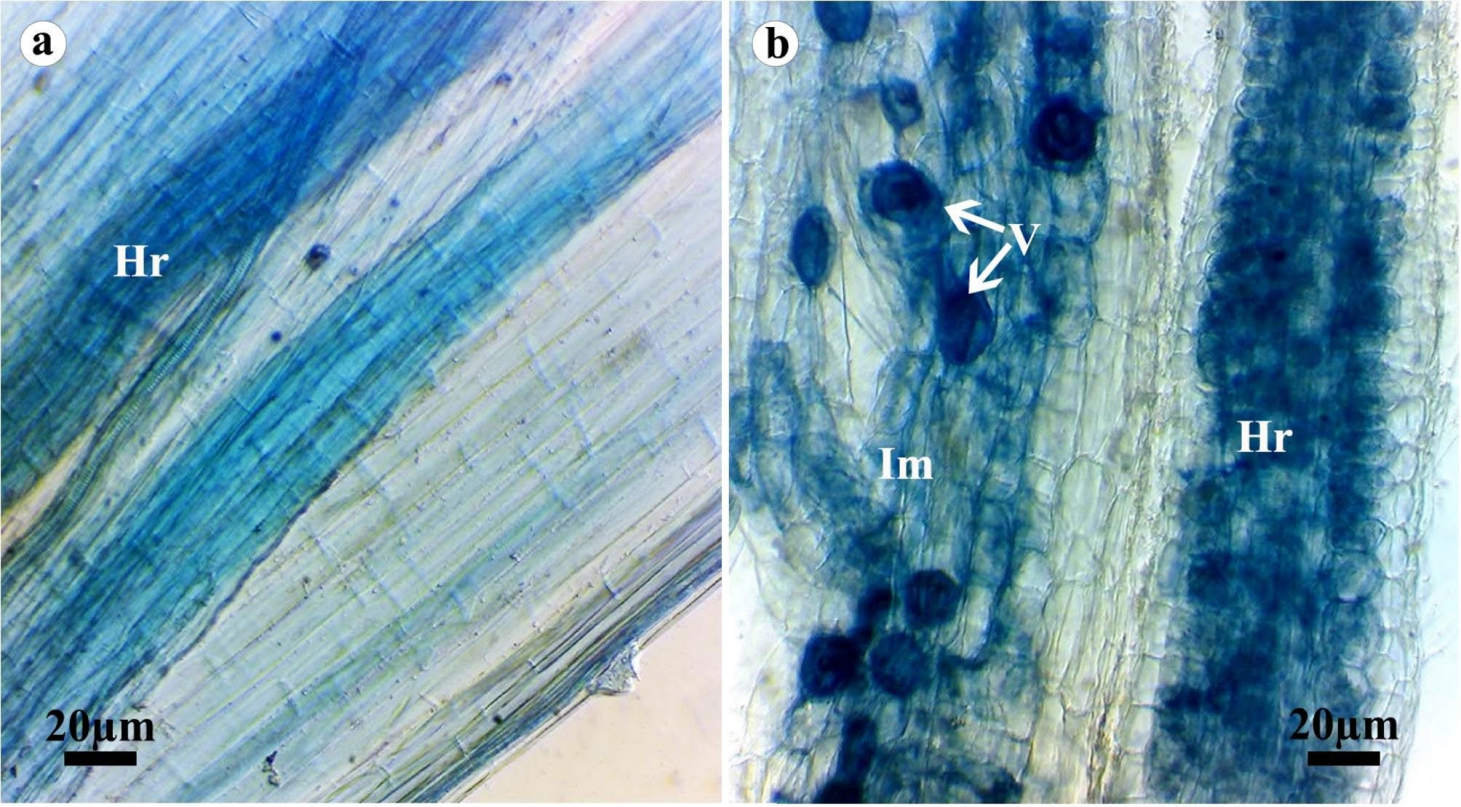
Microscopic images show mycorrhizal colonization in wheat roots, (**a**) non mycorrhizal root and (**b**) mycorrhizal roots, where Im: intraradical mycelium, V: vesicle, and Hr: host root

Mycorrhizal colonization in wheat plants infected with stripe rust in response to treating with *S. viridosporus* HH1 two weeks after infection is presented in Table 4; Fig. 8. Microscopic observations revealed that treatments (S + P) and (P) showed no mycorrhizal colonization, while the treatments inoculated with AMF (M + P) and (S + M + P) exhibited a considerable mycorrhization recording 52.2 and 55.4% colonization intensity, respectively. However, no significant difference was observed between the treatments (M + P) and (S + M + P) regarding the evaluated colonization parameters.

**Table 4:** Mycorrhizal colonization in wheat plants infected with stripe rust in response to treating with *Streptomyces viridosporus* HH1 two weeks after infection*

Treatment	Colonization Frequency (%)	Colonization Intensity (%)	Arbuscules Frequency (%)
P	0^b^	0^b^	0^b^
S + P	0^b^	0^b^	0^b^
M + P	63.1 ± 3.9^a^	52.2 ± 3.3^a^	32.6 ± 2.3^a^
S + M + P	66.2 ± 4.4^a^	55.4 ± 4.1^a^	34.5 ± 3.5^a^

* In each column, values followed by the same letter are not significantly different according to Tukey’s HSD test (*P* ≤ 0.05), each value is the mean of five replicates ± SD. Where, P: infected and non-treated, S + P: infected and sprayed with *S. viridosporus* HH1, M + P: infected and colonized with AMF, and S + M + P: infected, colonized with AMF, and sprayed with *S. viridosporus* HH1

## Discussion

In this study, the time-course changes in the transcriptomic expression of the polyphenol biosynthesis pathways genes in stripe rust-infected wheat plants were investigated. The defense mechanisms triggered by the mycorrhizal colonization and/or spraying with *S. viridosporus* HH1 were also studied. Polyphenols, particularly flavonoids, and chlorogenic acid, play important roles in enhancing the defense responses to different invading pathogens [17]. Results obtained in this work revealed that the dual treatment (S + M + P) had superiority over the single treatments in triggering the addressed genes, particularly at 3 dpi. At this time, ten of the thirteen genes were significantly upregulated, and most of them showed a very high significant upregulation as illustrated from the volcano plots. This result supports the synergism between the tested bio-agents and their overexpression of the studied genes, at varied degrees. In addition, it supports the significant reduction in the disease severity due to the dual treatment. Results from the qPCR in this study demonstrated that *C3H* was the highest upregulated gene (1977-fold), which indicated its importance as the probable main defense mechanism against *P. striiformis*. *C3H* has a pivotal role in the monolignols biosynthesis, the monomers of lignin molecule [18], and chlorogenic acid [19]. In this case, thickening of the cell wall via deposition of lignin leads to reinforcement of the cell wall rigidity and improvement of its imperviousness to act as a physical barrier to restrict penetration of *P. striiformis* and prevent nutrients transport from the cell cytoplasm to its haustorium and therefore starvation to death occurs [20]. *C3H* encodes an enzyme that catalyzes the key step (conversion of 4-coumarate to caffeate) in lignin and chlorogenic acid biosynthesis [18]. Anterola and Lewis [21] reported that downregulation of *C3H* resulted in a reduction in the lignin deposition. Induction of lignin deposition in the infected plant cells in response to mycorrhizal colonization has been widely reported [4, 22]. In addition to *C3H*, results from the present study showed that *HQT* and *HCT* were also overexpressed due to the dual treatment (S + M + P) at 3 dpi. *HQT* and *HCT* have key roles in the chlorogenic acid biosynthesis. *HQT* encodes for an enzyme that catalyzes the conversion of caffeoyl-CoA and quinic acid to chlorogenic acid, while *HCT* encodes for an enzyme that catalyzes the conversion of coumaroyl shikimate to caffeoyl-CoA [13]. Chlorogenic acid is a potent polyphenol, which acts as antioxidant (free radical scavenger) and plays an important role in the plant resistance against pathogenic fungi. Martínez et al. [23] reported that chlorogenic acid showed a full suppression of the mycelial growth and spore germination of various fungal pathogens. Results recorded in this study regarding upregulation of lignin and chlorogenic acid-related genes due to mycorrhizal colonization are in consistence with that obtained by Rashad et al. [24] on the mycorrhizal sunflower plants, which were infected with *Rhizoctonia solani*.

In the same time, data obtained in this study indicated the overexpression of the flavonoid biosynthesis pathway-related genes *CHS*, *CHI2*, *F3H, F3’H, DFR*, and *AN2* due to the dual treatment (S + M + P) at 3 dpi. *CHS* encodes for an enzyme that catalyzes the conversion of 4-coumaroyl-CoA to chalcone. It is considered the structural precursor and a pivotal intermediate for biosynthesis of many flavonoids, isoflavonoids, flavones, and anthocyanins [14]. In this concern, *CHI2* encodes for an enzyme that catalyzes conversion of chalcone to flavanone that can be converted to different flavonoids compounds. *F3H* and *F3’H* encode for enzymes that catalyze conversion of flavanone to form dihydroxyflavonol, and conversion of dihydroxyflavonol to dihydroquercetin, respectively [25]. In this regard, Hammerbacher et al. [26] found that *F3H* had a key role in the biosynthesis of multiple resistance-related polyphenolic compounds (taxifolin and catechin) in *Picea abies* against the bark beetle-associated fungus *Endoconidiophora polonica*. Furthermore, downregulation of this gene resulted in a significant reduction in production of dihydroflavonols and flavan-3-ols. *DFR* encodes an enzyme that catalyzes conversion of flavanone to flavan-4-ols, and the key enzyme, which catalyzes reduction of dihydroquercetin to anthocyanidin in the anthocyanin biosynthesis [15]. A downregulation of *DFR* in sweet potato was found to lead to a dramatic reduction in the anthocyanin production in their leaves and stem [27]. *AN2* is a MYB transcription factor-encoding gene, which coordinately regulates expression of the structural genes in anthocyanin biosynthesis pathway *PAL, CHS, CHI, F3H, F3’H*, and *DFR* [28]. In tobacco, upregulation of *AN2* was found to overexpress these structural genes in addition to *FLS, ANS, An1a*, and *An1b* genes and enhance anthocyanin accumulation [29]. Anthocyanins are flavonoid pigments, which have multiple physiological roles in the plant including the defense against different biotic and abiotic stresses [30]. Their fungitoxic potential was reported against different pathogenic fungi and overexpression of anthocyanin biosynthesis genes in response to plant fungal infection was also reported [31]. These results were supported by the high accumulation of the detected flavonoids and phenolic acids via HPLC analysis in this study, especially cinnamic acid (1000%), coumarin (488.23%), and esculetin (329.5%). Upregulation of lignin, chlorogenic acid, and flavonoid biosynthesis genes, which was reported in this study, indicates their contribution in the wheat resistance against *P. striiformis*, particularly in restricting penetration of its haustoria in their leaves cells in order to starve them to death. The obtained results are in agreement with that reported by El-Sharkawy et al. [16] who reported the biocontrol activity of mycorrhizal colonization and *S. viridosporus* HH1 against stripe rust in wheat plants. They recorded a triggering effect of these bioagents on expression of different defense-related genes and accumulation of the phenolic content as well as an increment of activity of various antioxidant enzymes.

Heatmap and hierarchical cluster analysis indicated that all applied treatments were clustered in two main groups, where all treatments at 1 dpi and treatment (P) at 3 dpi were categorized in one group and all other treatments in the second main group. One of the most interesting results obtained in the heatmap is the noticed downregulation of around half of the studied genes (six out of thirteen) in response to all treatments at 1 dpi and treatment (P) at 1 and 3 dpi (the black square). This phenomenon can be discussed in the light of the dominance of the pathogen effect in the first stages of infection, at which it intended to obstruct the plant resistance genes in order to make a successful﻿ penetration. In other words, the pathogen surprised the plant and prevented it to defend himself at this stage of infection. The same is valid for the treatment (P) at 3 dpi, in absence of the two-biocontrol agents (S) and (M). This may explain the fast spread of *P. striiformis* in the infected plant in a short period, especially in this susceptible cultivar (cv. Sids12). In contrast, the second group of the clusters included the other treatments where the applied biocontrol agents induced the plant resistance genes, at different degrees to fight the pathogen. Among the applied biocontrol treatments, the treatments (S + M + P) and (M + P) at 3 dpi were clustered in one sub-sub-cluster, and the treatments (S + M + P) and (M + P) at 7 dpi were clustered in the other sub-sub-cluster indicating the dominance of the mycorrhizal effect on that of *S. viridosporus* HH1 at both times. In the same time, grouping of those treatments at 3 and 7 dpi in one sub-cluster demonstrated that they were similar, to some extent, in their triggering effects on expression of the majority of the studied genes. On the other hand, downregulation of the six genes (*HQT*, *FLS1*, *AN2*, *F3’H*, *AN1*, and *CHS*) at the 1 dpi by all treatments resulted in their clustering in one main group, while all the other genes clustered in the other main group. Among all studied genes, the most frequent expressed gene in response to all applied treatments along the study period was *DFR.*

Pairwise correlation among the polyphenol biosynthesis pathways genes under the applied treatments revealed many significant correlations among them, at varied degrees. Among the noticed correlations, *AN2*-*FLS1*, *HQT-DFR*, and *HQT-HCT* were found positively correlated at a very high significance. This result indicates the coordination in their expression and the coherence in the total biosynthetic pathway in response to the tested treatments. The highly significant positive correlation between *HQT-HCT* is anticipated as they share the same pathway of chlorogenic acid biosynthesis. In the line with these results, Rashad et al. [24] observed a correlation between upregulation of different flavonoid biosynthesis genes and resistance of sunflower to *R. solani.* The high correlation between *AN2*-*FLS1* is also expected, where *AN2* is a transcription factor that regulates many genes in the flavonoid pathway including *FLS1.* Other high significant correlations, recorded in this study, between the studied genes in the flavonoid biosynthesis pathway indicate the coordination between them in this pathway.

The gene co-expression network was generated for the studied genes to detect which of them had a tendency to exhibit a coordinated expression pattern in these pathways in response to the applied treatments. Results obtained revealed that *HQT* was the most central gene with respect to the other genes. This means that it had the highest number and degree of significant correlations with the other genes, followed by *AN2*, and *DFR*, respectively. This indicates their key roles in the polyphenol biosynthesis pathways. One of the interesting results obtained from the co-expression network was the community which was formed by *DFR, HQT, HCT, F3H*, *F3’H*, *AN2, CHS*, and *FLS1.* This means that they have positive significant interrelationships with each other a way from *C3H, AN1, PAL1*, and *C4H*. In other words, when some genes show similar expression patterns, it means that they have a high probability of sharing the regulation mechanisms for their expression. On the contrary, *C3H* and *PAL1* were found to be peripheral in the co-expression network showing no significant correlation with the other genes, which indicates their independent expression.

## Conclusion

This study indicated that application of AMF and *S. viridosporus* HH1 synergistically triggered most of the polyphenol biosynthesis genes in wheat leaves against infection with *P. striiformis. C3H* was the most expressed gene indicating its importance as the main defense mechanism. Furthermore, most of the chlorogenic acid and flavonoid biosynthesis genes were also overexpressed. Up-regulation of these genes due to the treatment (S + M + P) enhanced resistance of wheat plants to stripe rust. Accumulation of different flavonoids and phenolic acids were detected in response to the dual treatment application, in particular, cinnamic acid, coumarin, and esculetin.

## Methods

### Wheat cultivar and microbial inocula

The wheat grains (cv. Sids12, bread wheat), used in this study, were obtained from Agricultural Research Centre, Egypt. Three types of AMF (in equal ratio) were utilized in the greenhouse experiment, namely *Rhizoglomus clarum* (Nicolson and Schenck) Sieverd., Silva and Oehl, *Gigaspora gigantea* (Nicol. and Gerd.) Gerd. and Trappe, and *Rhizophagus irregularis* (Blaszk., Wubet, Renker and Buscot) Walker and Schüßler. The AMF inoculum was obtained from Agricultural Research Centre, Egypt. The AMF were propagated under sudangrass (*Sorghum bicolor* L. Moench) for six months in a sterilized soil. The AMF inoculum (77% colonization index) was composed of spores, mycelia, root pieces, and rhizospheric soil.

The bacterial inoculum was prepared by culturing an isolate of *S. viridosporus* HH1, obtained from Agricultural Research Centre, Egypt, on nutrient broth (Biolab Zrt., Budapest, Hungary) at 37 °C for 10 days. The bacterial culture was used to prepare the inoculum at 10^6^ CFU mL^− 1^.

For the pathogen inoculum, a fresh spore suspension (10^5^ spore mL^− 1^) was prepared using uredospores of *P. striiformis* f. sp. *tritici*, obtained from Agricultural Research Center, Egypt. Tween 80 (0.5 mL L^− 1^) was added to the uredospore suspension as a surfactant agent to decrease clumping of the uredospores and enhance their dispersion on wheat leaves.

### Greenhouse experiment

Wheat grains were surface-sterilized using NaOCl solution (0.5%) and ethyl alcohol (70%) before sowing in pots (25 cm diameter) filled with sterilized soil at 5 grains per pot. For AMF application, 10 g of the mycorrhizal inoculum were added under each grain at the sowing time. No fertilization was applied in this experiment. For application of *S. viridosporus* HH1, some plants were sprayed with the bacterial inoculum 30 days after planting until run-off. Three days after application of the bacterial inoculum, all plants were sprayed with the uredospores suspension until run-off occurred. All plants were regularly irrigated. For each treatment, ten replicates were used. The complete randomized design was used to arrange the pots. The pots were preserved in a plastic hood at 20 °C and 85% humidity for two days, then kept in a greenhouse at 25/20°C (day/night) and 70% humidity. The tested treatments were as follows: infected and non-treated (P), infected and colonized with AMF (M + P), infected and sprayed with *S. viridosporus* HH1 (S + P), and infected, colonized with AMF, and sprayed with *S. viridosporus* HH1 (S + M + P).

### Transcriptomic patterns of the polyphenol biosynthesis pathways genes

Wheat leaves of the tested treatments were sampled 1, 3, and 7 (dpi). Total RNA of each sample was extracted using Spectrum Plant Total RNA Kit (Sigma-Aldrich, St. Louis, USA). cDNA was synthesized from the extracted RNA using High-Capacity cDNA Reverse Transcription Kit (Applied Biosystems, Foster City, USA) based on the manufacturer’s instructions. The PCR mixture composed of RNA (3 µL, 33 ng), dNTPs (2.5 µL, 11 mM), oligo (dT) primer (5 µL, 6 pmol µL^− 1^), 5X-buffer (2.5 µL), reverse transcriptase (Qiagen, Valencia, USA) (0.3 µL) and distilled water (6.7 µL). The amplification was occurred at 42 °C for 1 h and 95 °C for 10 min using a thermocycler (Promega, Germany).

The real-time PCR (qPCR) analysis was performed using CFX Connect Real-Time System (Bio-Rad, Feldkirchen, Germany). The qPCR mixture composed of cDNA (3 µL), SYBR Green Master Mix (Bioline, Germany) (12.5 µL), sterilized RNase-free water (1.5 µL), and 1.5 µL of each primer (forward and reverse, 12 pmol µL^− 1^). Sequences of 13 primers of the polyphenol biosynthesis pathways genes in wheat plants, used in this study, are presented in Table 5. Two reference genes, *α*-tubulin and *β*-actin, were used in this study due to their high stability during the mycorrhizal colonization [32]. The qPCR was performed as follows: a cycle at 95 °C for 3 min and 45 cycles (95 °C for 15 s, 56 °C for 30 s and 72 °C for 30 s). Each treatment was analyzed in triplicates (biological and technical). Relative expression levels were calculated using the comparative CT method (2^−ΔΔCT^) according to Livak and Schmittgen [33]. The expression values were then transformed into log_2_FC due to the overdispersion of the data (not normally distributed).

**Table 5:** Primer sequence of the studied polyphenol biosynthetic pathways genes

Primer name	Abbreviation		(5’-3’)	Product size (bp)	Melting Temp (°C)	Reference
Phenylalanine ammonia lyase 1	*PAL1*	F R	ACGGGTTGCCATCTAATCTGACA CGAGCAATAAGAAGCCATCGCAAT	92	80	AY005474
Cinnamic acid 4-hydroxylase	*C4H*	F R	CCCAGT TTTTGGAAATTGGCTTCA GCCCCATTCTAAGCAAGAGAACAT C	104	75	CK157495
Chalcone synthase	*CHS*	F R	CACCGTGGAGGAGTATCGTAAGGC TGATCAACACAGTTGGAAGGCG	93	82	EU408770
Chalcone isomerase 2	*CHI2*	F R	GGCAGGCCATTGAAAAGTTCC CTAATCGTCAATGATCCAAGCGG	103	78	AB187026
Flavanone 3-hydroxylase	*F3H*	F R	CCAAGGCATGTGTGGATATGGACC CCTGGATCAGTATGTCGTTCAGCC	103	78	AB187027
Flavonoid 3’-hydroxylase	*F3’H*	F R	TGGGTATACCCAAACTCATTCCG AAAAGCCCAAAGTTGATGTGAAAGG	96	77	AY519468
Dihydroflavonol-4-reductase	*DFR*	F R	TCACAGGAGCAGCTGGATTTATCG TCAGGATCACGAACAGTAGCATGG	91	79	AY209183
Flavonol synthase 1	*FLS1*	F R	CCTCCTTCCTACAGGGAAGCAAA CAAGCCCAAGTGACAAGCTCCTAA	91	79	KJ193852
Anthocyanin 1	*AN1*	F R	CCTCAACCTCAGAAATTCAGAAGC TCGTTGTTGTTGTCGTTCGATGC	102	75	MG979067
Anthocyanin 2	*AN2*	F R	ACAAGATGCCACTTTCCTTCACC TGTGCATCGTTGGGAGTTAGG	101	75	XM_044522067
Hydroxycinnamoyl-CoA quinate hydroxycinnamoyl transferase	*HQT*	F R	CCCAATGGCTGGAAGATTAGCTA CATGAATCACTTTCAGCCTCAACAA	99	76	OP295931
Hydroxycinnamoyl-CoA shikimate hydroxycinnamoyl transferase	*HCT*	F R	TCTCCAACCCCTTTTAACGAACC CAACTTGTCCTTCTACCACAGGGAA	103	80	OP295858
4-coumarate 3-hydroxylase	*C3H*	F R	TTGGTGGCTACGACATTCCTAAGG GGTCTGAACTCCAATGGGTTATTCC	100	82	CD895891
*α*-tubulin (Reference gene)	*α*-tubulin	F R	TATCTGCTACCAGGCTCCCGAGAA TGGTGTTGGACAGCATGCAGACAG	116	85	
*β*-actin (Reference gene)	*β*-actin	F R	GTGGGCCGCTCTAGGCACCAA CTCTTTGATGTCACGCACGATTTC	540	88	

### Disease assessment

Two weeks after infection, five wheat plants in each treatment were scored for the severity of stripe rust using a scale ranged between 1 and 100% [34]. The field response of the samples was evaluated according to Stakman et al. [35], where immune (I): no infection, resistant (R): small uredial pustules with necrosis, moderately resistant (MR): medium uredial pustules with necrosis, moderately susceptible (MS): large uredial pustules with chlorosis, and susceptible (S): large uredial pustules without necrosis or chlorosis).

### Estimation of colonization level

Two weeks after infection, five roots of wheat plants in each treatment were evaluated for the mycorrhizal colonization level. For each sample, wheat roots were cut into 1 cm pieces, treated with potassium hydroxide solution at 90 °C for 60 min, and stained with trypan blue. Using light microscope, frequency and intensity of root colonization, and frequency of arbuscules were determined [36].

### Profiling of selected phenolic acids and flavonoids

Ten selected phenolic acids and flavonoids were estimated in wheat leaves of the applied treatments using a high-performance liquid chromatography (HPLC) system (Agilent 1100, Agilent Technologies Inc., CA, USA). The chromatographic separation was performed using the column (Eclipse Plus C18, 4.6 mm x 150 mm, (5 µm particle size, Agilent Co.) at 1 mL min^-1^, 35°C, and UV 280 nm. A mixture standard solution contained ten selected phenolic acids and flavonoid compounds (gallic acid, protocatechuic acid, cinnamic acid, naphthylacetic acid, vanillic acid, 4,3-indole butyl acetic acid, catechin, esculetin, pyrocatechol, and coumarin) was used in this analysis.

### Statistical analyses

All statistical analyses were performed using R software version 4.2.2, where volcano plots were generated based on t-test using *ggplot2* package, gene co-expression network was generated using *qgraph* package based on Kendall’s Tau correlation coefficient, and the heatmap was produced using *pheatmap* package based on log_2_ fold change of gene expression [37]. Data were tested for their normality, and subjected to analysis of variance. Their means were statistically compared according to Tukey’s HSD test at *p* ≤ 0.05 using CoStat software version 6.4.

## Data Availability

The data that support the findings of this study are available from the corresponding author upon reasonable request.
